# Anti-inflammatory effects of *Flos Lonicerae Japonicae* Water Extract are regulated by the STAT/NF-κB pathway and HO-1 expression in Virus-infected RAW264.7 cells

**DOI:** 10.7150/ijms.56198

**Published:** 2021-03-30

**Authors:** Hui-Wen Lin, Yi-Ju Lee, Deng-Jye Yang, Ming-Chang Hsieh, Ching-Chung Chen, Wei-Li Hsu, Yuan-Yen Chang, Cheng-Wei Liu

**Affiliations:** 1 Department of Optometry, Asia University, Taichung, Taiwan.; 2 Genetics Center, Department of Medical Research, China Medical University Hospital, and School of Chinese Medicine, China Medical University, Taichung, Taiwan.; 3 Department of Pathology, Chung-Shan Medical University; Department of Pathology, Chung-Shan Medical University Hospital, Taichung, Taiwan.; 4 Institute of Food Safety and Health Risk Assessment, National Yang‐Ming University, Taipei, Taiwan.; 5 Department of Medical Laboratory and Biotechnology, Chung Shan Medical University; Clinical Laboratory, Chung Shan Medical University Hospital, Taichung, Taiwan.; 6 Graduate Institute of Microbiology and Public Health, College of Veterinary Medicine, National Chung Hsing University, Taichung, Taiwan.; 7 Department of Microbiology and Immunology, School of Medicine, and Chung Shan Medical University; Clinical Laboratory, Chung Shan Medical University Hospital, Taichung, Taiwan.; 8 Department of Post-Modern Agriculture, MingDao University, Changhua, Taiwan.

**Keywords:** Flos Lonicerae Japonicae water extract (FLJWE), pseudorabies virus (PRV), antiviral inflammatory, heme oxygenase-1 (HO-1)

## Abstract

This study examined the effect of the Flos Lonicerae Japonicae water extract (FLJWE), chlorogenic acid, and luteolin on pseudorabies virus (PRV)-induced inflammation in RAW264.7 cells and elucidated related molecular mechanisms. The results revealed that FLJWE and luteolin, but not chlorogenic acid, inhibited the production of inducible nitric oxide synthase (iNOS), cyclooxygenase-2 (COX-2), and inflammatory cytokines in PRV-infected RAW 264.7 cells. We found that the FLJWE and luteolin suppressed nuclear factor (NF)-κB activation by inhibiting the phosphorylation of signal transducer and activator of transcription 1 and 3 (STAT1 and STAT3, respectively). Moreover, the FLJWE significantly upregulated the expression of pNrf2 and its downstream target gene heme oxygenase-1 (HO-1). Our data indicated that FLJWE and luteolin reduced the expression of proinflammatory mediators and inflammatory cytokines, such as COX-2 and iNOS, through the suppression of the JAK/STAT1/3-dependent NF-κB pathway and the induction of HO-1 expression in PRV-infected RAW264.7 cells. The findings indicate that the FLJWE can be used as a potential antiviral agent.

## Introduction

Flos Lonicerae Japonicae (FLJ), also commonly referred to as honeysuckle, is the flower bud of *Lonicera japonica* Thunb. (*Caprifoliaceae*) 1. FLJ has long been used in Chinese herbal medicine because of its antibacterial, antiviral, anti-inflammatory, antioxidative, and hepatoprotective properties 2-5, which may be associated with its active components such as organic acids, flavones, and saponins).

FLJ is a traditional herbal medicine that has been widely used in East Asia as an anti-inflammatory and antioxidative agent. Studies have reported that the FLJ extract exerted a strong anti-inflammatory effect and could effectively inhibit bacterial lipopolysaccharide (LPS)-induced inflammatory mediators *in vitro* 4,6. In addition, these studies have suggested that FLJ could inhibit the responses of various inflammatory factors, such as cyclooxygenase- 2 (COX-2), tumor necrosis factor-α (TNF-α), and interleukin-6 (IL-6), by regulating the phosphorylation of phosphoinositide 3-kinase (PI3K)/Akt and mitogen-activated protein kinases (MAPKs).

In the present study, we evaluated whether organic FLJ flowers, which are suited to the local environment of Taiwan, would be beneficial in producing a pollutant-free, high-quality FLJ water extract (FLJWE). These plants were grown and subjected to quality control according to the guidelines of organic farming and the global good agriculture practice (GAP) system. In addition, we analyzed the composition and biological activity of the FLJWE.

Our previous studies have reported that RAW264.7 cells (macrophage-like cell line from a mouse) could be used as an *in vitro* screening platform for pseudorabies virus (PRV)-induced inflammation 7-10. The main aim of this study was to investigate the anti-inflammatory effect of the FLJWE on virus-induced inflammation.

Inflammation at the site of injury or infection is one of the earliest biological functions suggested to be mediated by the phosphorylation of STAT proteins 11-14. The preferential activation of signal transducer and activator of transcription 1 and 3 (STAT1 and STAT3, respectively) was observed in response to the release of endogenous IL-6 within stimulated cells 15, 16. However, the molecular mechanisms underlying the anti-inflammatory effect of the FLJWE on virus-induced inflammation remain unclear.

Heme oxygenase-1 (HO-1), an inducible, anti-inflammatory, and immunosuppressive enzyme, could protect against apoptosis, inflammation, and oxidative stress *in vitro* 17, 18. A study reported that HO-1 attenuated inflammation and modulated immune responses both *in vitro* and *in vivo* 19. Nuclear factor erythroid-2-related factor 2 (Nrf2) is the main transcription factor that induces the expression of HO-1 and other antioxidant enzymes 20, 21. Therefore, in this study, we elucidated the mechanism through which the FLJWE resulted in the expression of HO-1 and pNrf-2 (Nrf2 activation) in PRV-infected RAW264.7 cells.

Phenolics and flavonoids play key roles in the antioxidative activity of plants 22-25. In this study, we first evaluated the antioxidative and anti-inflammatory properties of the FLJWE by examining their radical scavenging activity and total phenolic concentration. Furthermore, we examined whether the anti-inflammatory effect was induced by the production of inducible nitric oxide synthase (iNOS), COX-2, and inflammatory cytokines, such as IL-6, TNF-α, RANTES, and monocyte chemoattractant protein-1 (MCP-1), through the inactivation of NF-κB and STAT1/3 and the activation of HO-1 in PRV-infected RAW264.7 macrophages.

## Materials and methods

### Chemicals and reagents

Potassium hydroxide (KOH) was purchased from Merck (Darmstadt, Germany). The following reagents and chemicals were procured from Sigma (St. Louis, MO, USA): glutamine, 3-(4,5-dimethylthiazol-2-yl)-2, penicillin, streptomycin, phosphate-buffered saline, ethylenediaminetetraacetic acid, phenylmethanesulphonyl fluoride, sodium chloride, Tris, and Triton X-100. Fetal bovine serum (FBS) and RPMI-1640 medium were purchased from Gibco/Invitrogen (Carlsbad, CA, USA). In addition, specific antibodies for COX-2, iNOS, NF-κB, and β-actin were obtained from Santa Cruz Biotechnology (Santa Cruz, CA, USA). Enzyme-linked immunosorbent assay (ELISA) kits used to measure the levels of TNF-*α*, IL-6, MCP-1, and RANTES were procured from R&D Systems (Minneapolis, MN, USA). Horseradish peroxidase-conjugated antigoat, antirabbit, and mouse immunoglobulin G were purchased from Bio-Rad Laboratories (Hercules, CA, USA). Antibodies against STAT1/3 and pSTAT1/3 were obtained from Cell Signaling Technology (Danvers, MA, USA).

### Preparation of the FLJWE

*L. japonica* Thunb. was planted in the experimental farm of Ming Dao University (Changhua, Taiwan). Organic farming methods were used to manage and cultivate the plants. Plant production and quality control were in accordance with organic farming and global GAP protocols. We harvested flower buds at different stages from July to September in 2014. The concentrations of 398 pesticides (Appendix) in FLJ were analyzed using an AOAC Official Method of Analysis (SGS Taiwan Ltd.). We determined that the analyzed samples were free from the 398 pesticides. A total of 500 g of samples was collected for each group according to the developmental characteristics of the FLJ flower and then dried at 40°C for 96 h until fully dehydration. They were then ground into fine powder, which was stored in a cabinet drier until use in further experiments. Using water as a solvents, we obtained 15 g/L of the FLJWE by steeping a suitable amount of dried FLJ in boiled distilled water (100 °C) for 30 min before filtration (ADVANTEC No. 1). The water extract samples were stored at -20 °C.

### Total polyphenolic profile of the FLJWE

We spectrophotometrically examined the concentrations of total flavonoids and phenolic compounds in the FLJWE following a previous method 9,11. Phenolic acid compounds, namely gallic acid, gentisic acid, chlorogenic acid, p-hydroxybenzoic acid, vanillic acid, caffeic acid, p-coumaric acid, ferulic acid, sinapic acid, syringic acid, p-anisic acid, and rosmarinic acid, and flavonoid standards, namely those of catechin, rutein, quercitrin, luteolin, rosmaric acid, neohesperidin, hesperidin, morin, eriodictyol, daidzein, glycitrin, quercetin, diosmin, naringenin, genistein, nesperetin, apigenin, kaempferol, and isorhamnetin, were purchased from Sigma. The aforementioned phenolic acids and flavonoid compounds in the FLJWE were identified through high-performance liquid chromatography (HPLC) based on UV absorbance and retention time and quantified using the standard curves of authentic compounds.

### Cell line and virus

Murine macrophage RAW264.7 cells were obtained from the American Type Culture Collection (Manassas, VA, USA). These cells were cultured in RPMI-1640 medium supplemented with antibiotics (100 units/mL of penicillin and 100 μg/mL of streptomycin), 2 mM glutamine, and 10% heat-inactivated FBS in a humidified incubator (Astek Co. Fukuoka, Japan) containing 5% CO_2_ at 37 °C. The stock of PRV (strain of TNL) used in this study was amplified from PK15 cells; in addition, the titer was determined by performing a standard plaque assay in PK15 cells, as previously described 9, 10.

### Cell viability assay

RAW264.7 cells were seeded into 24-well plates at 1 × 10^6^ cells/well and incubated for 24 h; these cells were subjected to a 1.5-h preincubation process at different FLJWE concentrations (2.5, 5, 10, 20, and 40 μg/mL). Cell viability was measured using blue formazan, the metabolized product of 3-(4, 5-dimethylthiazol-2-yl)-2, 5-diphenyltetrazolium bromide) (MTT); blue formazan is activated by mitochondrial dehydrogenases only in live cells 9, 10.

### Determination of proinflammatory cytokines

RAW264.7 cells were pretreated under various conditions for 24 h. ELISA was performed to measure the levels of RANTES, IL-6, TNF-*α* and MCP-1 in the culture medium (supernatant) according to manufacturer protocols 9, 10.

### Western blotting

RAW264.7 cells were pretreated under different conditions for 24 h. After the incubation of cell lysates with specific antibodies, immunoblotting was performed, as described previously 9, 10.

### Statistical analysis

All experiments were performed in triplicate, and mean values were calculated. Analysis of variance was used to evaluate data, and differences in the mean ± standard deviation were determined using Duncan's multiple range test. The significance level was set at *p* < 0.05.

## Results and discussion

### Polyphenolic profile of the FLJWE

**Figure 1:**
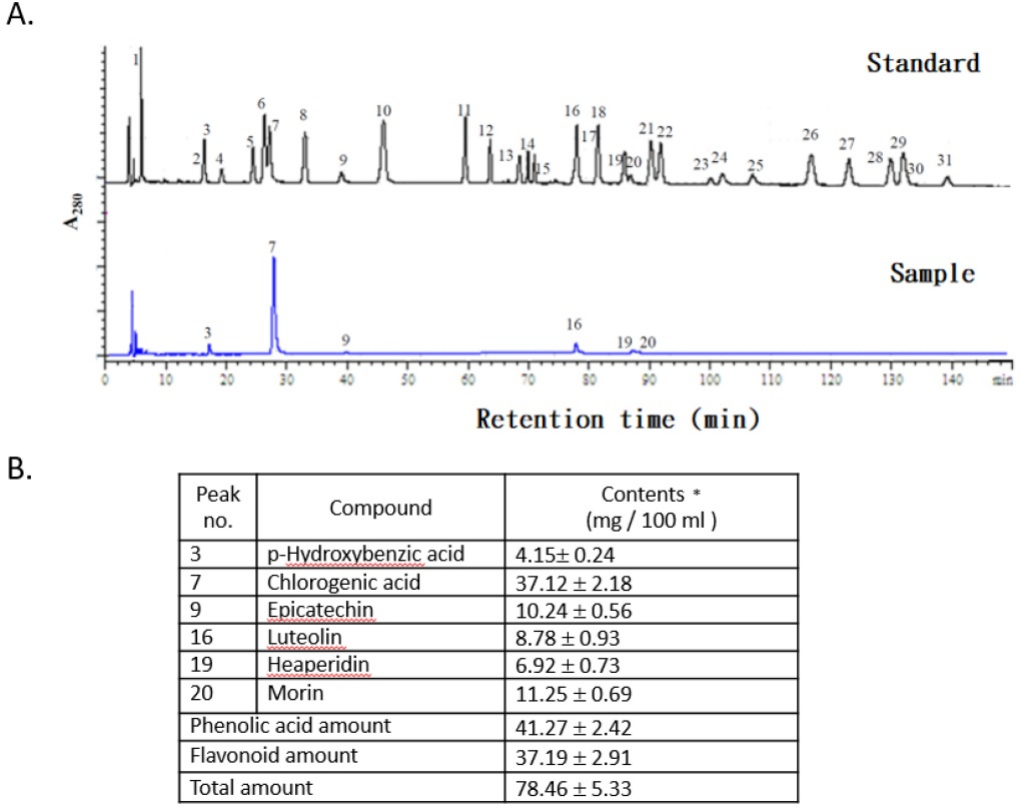
HPLC chromatograms of standards and the FLJWE. Peaks: (1) gallic acid, (2) gentisic acid, (3) *p*-hydroxybenzoic acid, (4) catechin, (5) vanillic acid, (6) caffeic acid, (7) chlorogenic acid, (8) syringic acid, (9) epicatechin, (10) *p*-coumaric acid, (11) ferulic acid, (12) sinapic acid, (13) *p*-anisic acid, (14) rutein, (15) quercitrin, (16) luteolin, (17) rosmaric acid, (18) neohesperidin, (19) hesperidin, (20) morin, (21) eriodictyol, (22) daidzein, (23) glycitrin, (24) quercetin, (25) diosmin, (26) naringenin (27) genistein, (28) nesperetin, (29) apigenin, (30) kaempferol, and (31) isorhamnetin. Data are presented as mean ± standard error of mean (n = 3).

The concentrations of phenolic acids and flavonoid compounds in the FLJWE were determined through HPLC (Fig. 1A). The concentrations of phenolic acids and total flavonoids in the FLJWE were approximately 41.27 ± 2.42 and 37.19 ± 2.91 mg per 100 mL of extract, respectively (Fig. 1B). Previous studies have identified the bioactive markers of chlorogenic acid and luteolin in FLJ 26, 27. The concentrations of chlorogenic acid and luteolin were 37.12 ± 2.18 and 8.78 ± 0.93 mg per 100 mL of extract, respectively. Furthermore, the concentrations of p-hydroxybenzoic acid, epicatechin, hesperidin, and morin were 4.15 ± 0.24, 10.24 ± 0.56, 6.92 ± 0.73, and 11.25 ± 0.69 mg per 100 mL of extract, respectively (Fig. 1B). These findings indicated that among phenolic acids, the proportion of chlorogenic acid (89.94%) was found to be the highest in the FLJWE. Phenolics and flavonoids play key roles in the antioxidative activity of plants 22-23, 28. In addition, chlorogenic acid and luteolin could activate Nrf2 in *in vitro* models, resulting in the upregulation of HO‐1 and the attenuation of excessive inflammatory responses 20, 29. Moreover, recent studies have indicated that epicatechin 30, hesperidin 31, and morin 32 exerted an anti-inflammatory effect on LPS-stimulated cells. On the basis of the antioxidative and anti-inflammatory properties of the major phenolic acids and flavonoid compounds, we investigated the *in vitro* effect of the FLJWE on virus-induced inflammation.

### Cytotoxicity of the FLJWE in RAW264.7 cells

**Figure 2:**
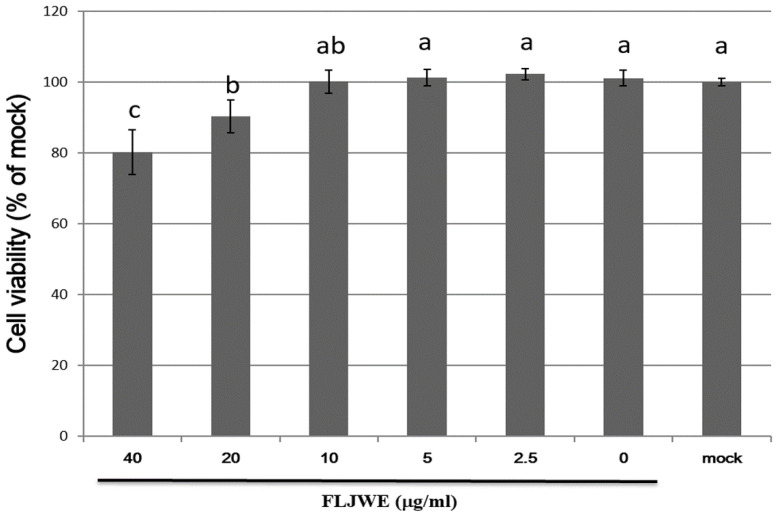
Cell viability of RAW264.7 cells treated with various concentrations of the FLJWE. RAW264.7 cells were treated with various concentrations of FLJWE, and their cell viability was examined using an MTT assay. Data are presented as mean ± SD (n = 3). Mean values with different letters are significantly different (p < 0.05).

We performed an MTT assay to examine the cytotoxic effects of the FLJWE at concentrations ranging from 2.5 to 40 μg/mL on the viability of RAW264.7 cells. The results revealed that an FLJWE concentration of <10 μg/mL exerted no toxic effects (p > 0.05) on RAW264.7 cells (Fig. 2). Therefore, we used the FLJWE concentrations of 2.5, 5 and 10 μg/mL in subsequent experiments.

### Effect of the FLJWE on proinflammatory cytokine production in RAW264.7 cells infected with PRV

**Figure 3:**
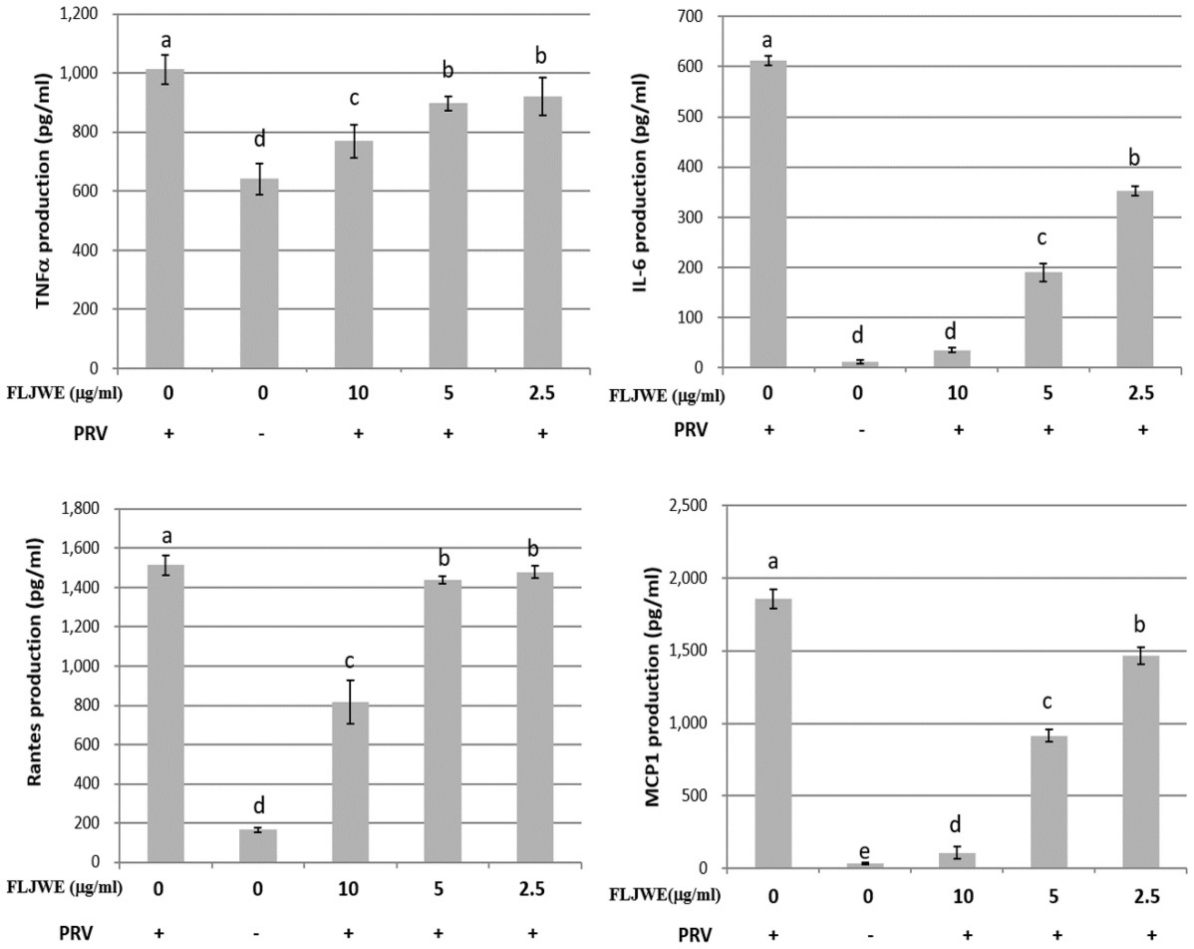
Inhibitory effects of the FLJWE on IL-6, MCP-1, RANTES, and TNF-α production in PRV-infected RAW264.7 cells. RAW264.7 cells were not treated or pretreated with various concentrations of the FLJWE (10, 5, and 2.5 μg/mL) for 1.5 h and then infected or not infected (control) with PRV (at an MOI of 0.1). After 24 h, the cultured medium was assayed to determine the levels of TNF-α, IL-6, RANTES, and MCP-1 through an enzyme-linked immunosorbent assay. The results are presented as mean ± SD (n = 3). Mean values with different letters are significantly different (p < 0.05).

The proinflammatory cytokines RANTES, MCP-1, IL-6, and TNF-α are involved in the immunopathology of acute or chronic inflammatory diseases 4, 9, 33. Therefore, we investigated the effect of the FLJWE on proinflammatory cytokine secretion in PRV-infected RAW264.7 cells. The cells were preincubated in the presence or absence of the FLJWE for 1.5 h before PRV infection (at a multiplicity of infection of 0.1). The cytokine concentration was measured using a commercially available ELISA kit. The results revealed that the FLJWE dose-dependently suppressed (p < 0.05) the levels of TNF-α, IL-6, RANTES, and MCP-1 secreted in PRV-infected RAW264.7 cells (Fig. 3).

### Effect of the FLJWE on NF-κB, COX-2, and iNOS expression in RAW264.7 cells infected with PRV

**Figure 4:**
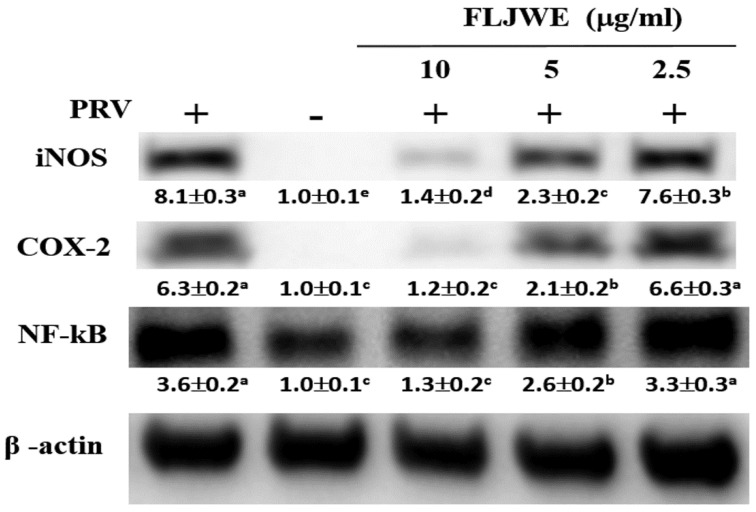
Effect of the FLJWE on iNOS, COX-2, and NF-κB expression in PRV-infected RAW264.7 cells. RAW264.7 cells were not treated or pretreated with various concentrations of the FLJWE (2.5, 5, and 10 μg/mL) for 1.5 h and then infected or not infected (control) with PRV (at an MOI of 0.1) for 24 h. All proteins were subjected to 10% SDS-PAGE followed by Western blotting with iNOS, COX-2, NF-κB, and β-actin antibodies. Results are presented as mean ± SD (n = 3). Mean values with different letters are significantly different (p < 0.05).

Our previous study revealed that PRV infection activates signal transduction, including NF-κB and MAPK cascades 8-10. NF-κB is a well-known transcription factor that induces the expression of proinflammatory genes such as iNOS and COX-2. To confirm that the FLJWE exerted an inflammatory effect on virus-induced inflammation, the expression of inflammation-related proteins, namely iNOS, COX2, and NF-κB, was examined through Western blotting. In the experiment, RAW264.7 cells were either infected with PRV or not. The expression of iNOS, COX2, and NF-κB proteins was significantly increased in the PRV-infected group compared with the uninfected group and was significantly suppressed by the FLJWE at a concentration of 10 μg/mL. The FLJWE dose-dependently inhibited the PRV-induced expression of NF-κB, COX-2, and iNOS (Fig. 4). These findings indicated that the FLJWE might exert an anti-inflammatory effect on virus-induced inflammation.

### Effect of the FLJWE on STAT1 and STAT3 expression in RAW264.7 cells infected with PRV

**Figure 5:**
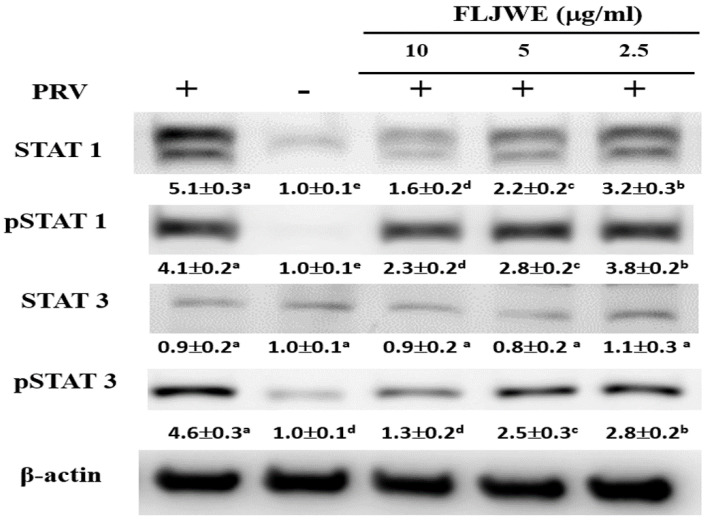
Effect of FLJWE on the expression of the STAT1/3 pathway in PRV-infected RAW264.7 cells. RAW264.7 cells were not treated or pretreated with various concentrations of the FLJWE (2.5, 5, and 10 μg/mL) for 1.5 h and then infected (control) or not infected with PRV (at an MOI of 0.1) for 24 h. All proteins were subjected to 10% SDS-PAGE followed by Western blotting with STAT1/3, pSTAT1/3, and β-actin antibodies. Results are presented as mean ± SD (n = 3). Mean values with different letters are significantly different (p < 0.05).

Recent studies have indicated that LPS induces IL-6 production by activating STAT1 and STAT3 signaling pathways 9, 33. Phosphorylated STAT dimers are major transcription factors that lead to inflammatory responses. Our previous study indicated that luteolin attenuated inflammatory responses through the suppression of the STAT1/3-dependent NF-κB pathway in PRV-infected RAW264.7 cells 10. Therefore, we examined whether the FLJWE can modulate PRV-induced STAT1 and STAT3 activation. As shown in Fig. 5, PRV infection considerably increased STAT1 and STAT3 phosphorylation compared with in the control condition. However, pretreatment with the FLJWE dose-dependently reduced PRV-induced STAT1 and STAT3 phosphorylation.

### Effect of the FLJWE on the expression of HO-1 and pNrf2 in PRV-infected RAW264.7 cells

**Figure 6:**
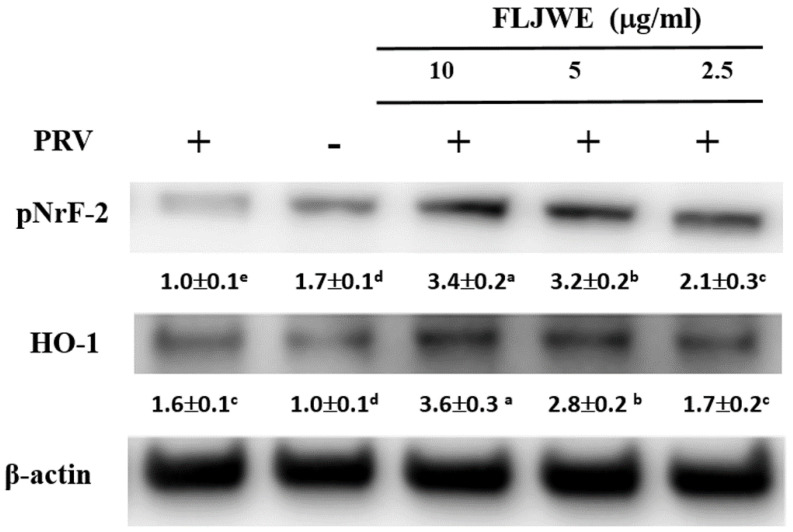
Effect of FLJWE on the expression of HO-1 and pNrf2 in PRV-infected RAW264.7 cells. RAW264.7 cells were not treated or pretreated with various concentrations of the FLJWE (2.5, 5, and 10 μg/mL) for 1.5 h and then infected or not infected with PRV (at an MOI of 0.1) for 24 h. Total protein was subjected to 10% SDS-PAGE followed by Western blotting with HO-1, pNrf2, and β-actin antibodies. Results are presented as mean ± SD (n = 3). Mean values with different letters are significantly different (p < 0.05).

HO-1 is a member of the heat-shock protein family; it is a crucial antioxidant and anti-inflammatory protein and is regulated by the activation of the major transcription factor Nrf2 34. HO-1 and its end products possess antioxidative, anti-inflammatory, antiapoptotic, and antiproliferative 35 and antiviral 36, 37 properties. Therefore, we evaluated whether the FLJWE exerts anti-inflammatory effects by inducing the expression of HO-1 and pNrf2 (Nrf2 activation). As shown in Fig. 6, PRV infection considerably reduced the phosphorylation of Nrf2 (pNrf2) compared with in the control condition. However, compared with the positive control (PRV-infected group), pretreatment with the FLJWE upregulated the protein levels of pNrf2 and HO-1. These results suggest that the FLJWE induces HO-1 in an Nrf2-dependent manner.

### Effect of the FLJWE and a comparison of the effects of luteolin and chlorogenic acid on proinflammatory cytokine production in PRV-infected RAW264.7 cells

**Figure 7:**
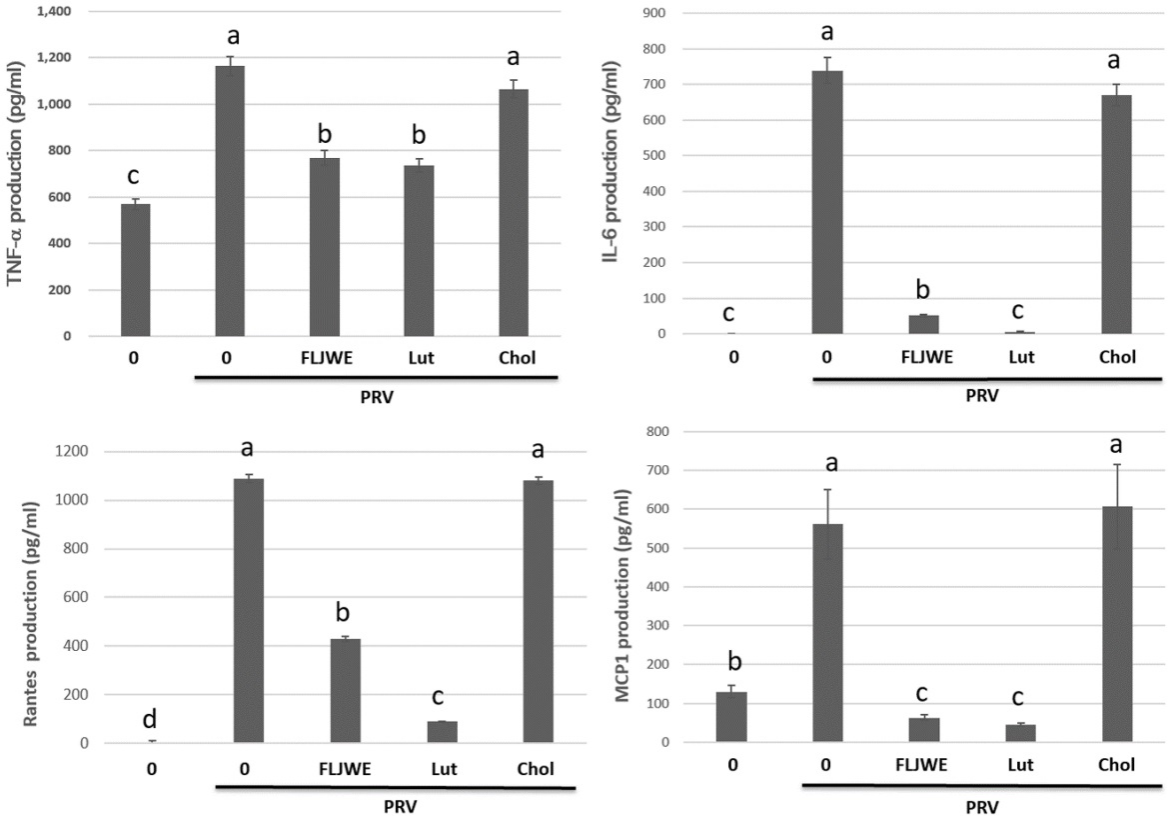
Effect of FLJWE, chlorogenic acid, and luteolin on inflammatory cytokines. RAW264.7 cells were not treated (control and PRV group) or pretreated with various concentrations of FLJWE, chlorogenic acid (Chol; 250 μM), or luteolin (Lut; 10 μM) for 1.5 h and then infected or uninfected (control) with PRV (at an MOI of 0.1). After 24 h, the cultured medium was assayed to determine the level of IL-6, MCP-1, RANTES, and TNF-α, which were measured using an enzyme-linked immunosorbent assay. Results are presented as mean ± SD (n = 3). Mean values with different letters are significantly different (p < 0.05).

Previous studies have examined the concentrations of chlorogenic acid and luteolin as standard chemicals to evaluate the chemical activity of FLJ 4, 38. Therefore, we investigated the anti-inflammatory effects of the FLJWE, chlorogenic acid, and luteolin on infected RAW264.7 cells. The cells were preincubated in the presence or absence of the FLJWE, chlorogenic acid (250 μM), and luteolin (10 μM) for 1.5 h before PRV infection. The cytokine concentration was measured using a commercially available ELISA kit. The findings indicated that the FLJWE and luteolin considerably inhibited the production of TNF-α, IL-6, MCP-1, and RANTES in infected RAW264.7 cells. However, chlorogenic acid did not effectively inhibit the production of various cytokines (Fig. 7).

### Effect of FLJWE and comparison of the effects of chlorogenic acid and luteolin on NF- κB, COX-2, and iNOS expression in PRV-infected RAW264.7 cells

**Figure 8:**
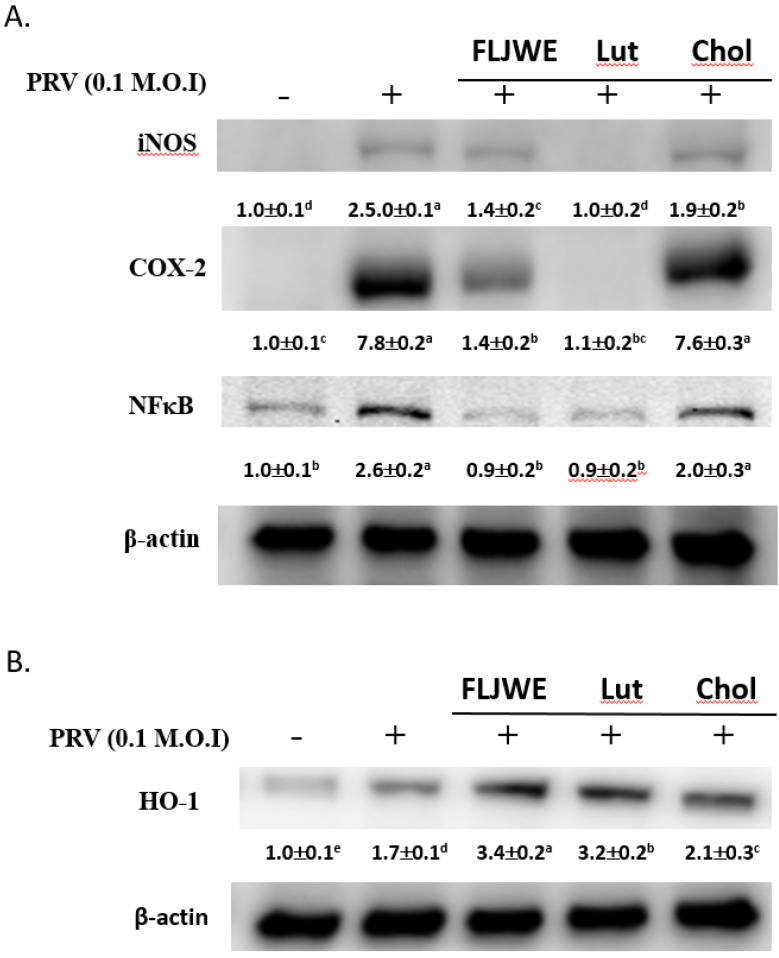
Comparison of the effects of FLJWE, chlorogenic acid, and luteolin on iNOS, NF-κB, and HO-1 expression in PRV-infected RAW264.7 cells. RAW264.7 cells were not treated (control and PRV group) or pretreated with various concentrations of FLJWE, chlorogenic acid (Chol; 250 μM), or luteolin (Lut; 10 μM) for 1.5 h and then infected or not infected (control) with PRV for 24 h. All proteins were subjected to 10% SDS-PAGE followed by Western blotting with iNOS, NF-κB, HO-1, and β-actin antibodies. Mean values with different letters are significantly different (p < 0.05).

We evaluated the regulation of iNOS, COX-2, and NF-κB expression by the FLJWE, chlorogenic acid, and luteolin in PRV-infected RAW264.7 cells. The results indicated that the FLJWE and luteolin, but not chlorogenic acid, strongly inhibited the expression of NF-κB, iNOS, and COX-2 in PRV-infected RAW264.7 cells (Fig. 8).

Our previous studies have reported that FLJWE could induce HO-1 expression. Thus, we subsequently compared the regulatory effects of FLJWE, chlorogenic acid, and luteolin on HO-1 expression in infected RAW264.7 cells. Our results indicated that FLJWE and luteolin strongly upregulated HO-1 expression in these cells. However, chlorogenic acid could only slightly induce HO-1 expression (Fig. 8B).

## Conclusions

More than 212 chemical compounds have been isolated from FLJ, and most of these are phenolic acids (e.g., chlorogenic acid, isochlorogenic acid, caffeic acid, and hexadecanoic acid), essential oils, flavones (e.g., quercetin, luteolin, and hyperoside), iridoids, saponins, and trace minerals 1, 38, 39,40. Zhang et al. (2011) evaluated the correlation between the antioxidative and anti-inflammatory activity of 14 Chinese medicinal plants by using water and ethanol extracts, and they reported that the high antioxidative activity of the extracts significantly contributed to the anti-inflammatory activity of these medicinal herbs. In addition, a study indicated that plant extracts containing high concentrations of phenolic acids and flavonoids exhibited substantial anti-inflammatory activity, resulting in high cell viability 22. According to the results of the aforementioned studies, the antioxidative activity of herbs can be attributed to their total concentrations of flavonoids and phenolic acids. Many studies have reported that extracts exhibiting strong antioxidative activity significantly contributed to the anti-inflammatory activity of medicinal herbs. The results of this study demonstrated that the FLJWE contains many polyphenols, with chlorogenic acid and p-hydroxbenzoic acid being the major phenolic acids and epicatechin, luteolin, hesperidin, and morin being the major flavonoids (Fig. 1B).

The experimental results indicated that the FLJWE exerted an anti-inflammatory effect on virus-induced inflammation by inhibiting iNOS, COX-2, and NF-κB (Fig. 4) and cytokines such as TNF-α, MCP-1, RANTES, and IL-6 (Fig. 3). A previous study reported that FLJ could attenuate LPS-induced inflammatory effects through NF-κB and MAPK (Cheng *et al.*, 2014) or IRAK-1/TAK1 and TBK1/IRF3 41 signaling pathways in RAW 264.7 macrophages. However, whether FLJWE can inhibit STAT/NF-κB-related inflammation in macrophages (RAW264.7 cells) infected with PRV remains unclear. In this study, we found that FLJWE could reduce the secretion of inflammatory cytokines (Fig. 3), and this might have further suppressed the activation of STAT1 and STAT3 in PRV-induced RAW264.7 cells (Fig. 5). This result is consistent with that of a previous study that reported that luteolin inhibits the phosphorylation of STAT1 and STAT3 (Liu et al., 2016). Furthermore, the results of this study indicated that luteolin attenuated inflammatory responses by inducing Nrf2-mediated HO-1 expression and by suppressing the STAT1/3-dependent NF-κB pathway in PRV-infected RAW264.7 cells.

HO-1 has been reported to exert antiviral effects on herpes simplex virus type 2 42, bovine viral diarrhea virus 43, and porcine reproductive and respiratory syndrome virus 44 *in vitro*. We examined the regulatory effect of the FLJWE on the HO-1/Nrf2 signaling pathway in PRV-infected RAW264.7 cells. Our data indicated that the FLJWE significantly upregulated the protein expression of pNrf2 and its downstream target gene HO-1 (Fig. 6). Our results indicated that FLJWE and luteolin strongly upregulated HO-1 expression in PRV-infected RAW264.7 cells. However, chlorogenic acid could only slightly induce HO-1 expression (Fig. 8B).

We observed that the major bioactive component of the FLJWE, chlorogenic acid, could not effectively inhibit virus-induced inflammatory responses (Figs. 7 and 8). This result is consistent with that reported by Jin *et al.* (2006) who indicated that chlorogenic acid (100 μg/mL of approximately 282 μM) did not exert an inhibitory effect on the levels of inflammatory mediators or the expression of COX-2 and iNOS in LPS-treated RAW 264.7 cells 39. Shen *et al.* (2017) reported that up to 1 mM chlorogenic acid could inhibit the LPS-induced expression of NO and IL-1β by inhibiting JAK2/STAT3 activation in RAW264.7 cells 45. These findings indicate that luteolin is the active substance component present in the FLJWE that exerted the anti-inflammatory effect on PRV virus-induced inflammatory responses.

**Figure 9:**
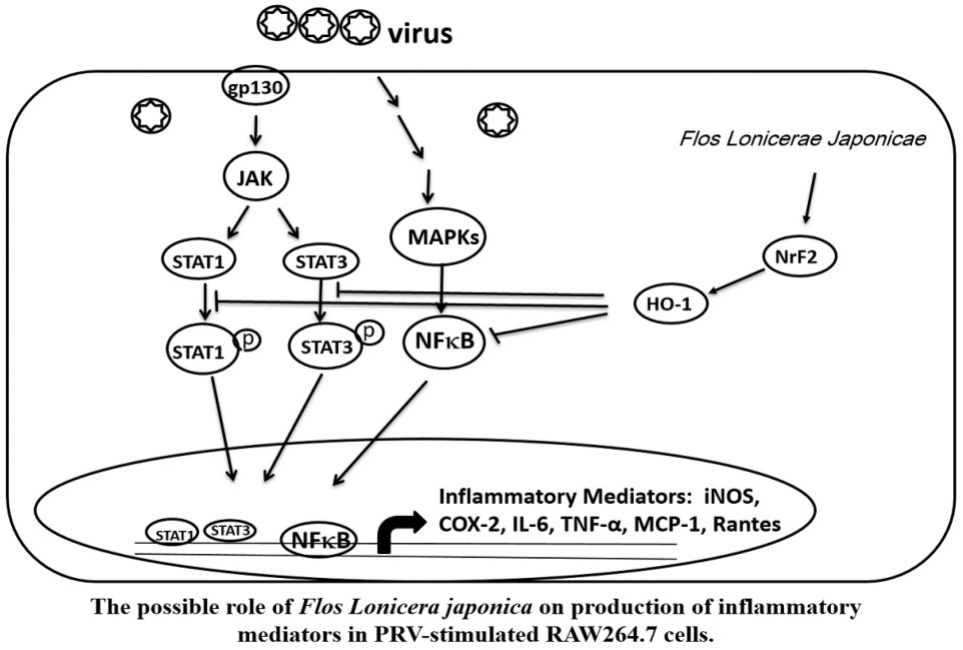
Possible role of *Flos Lonicerae Japonicae* in the production of inflammatory mediators in PRV-stimulated RAW264.7 cells.

Taken together, our data indicate that the FLJWE exerted an antiviral effect on PRV-infected RAW264.7 cells by reducing the expression of proinflammatory mediators, such as COX-2 and iNOS and inflammatory cytokines, such as TNF-α, MCP-1, RANTES, and IL-6, through the suppression of the JAK/STAT1/3-dependent NF-κB pathway and the induction of HO-1 expression (Fig. 9). The findings of this study provide a pharmacological basis for using FLJWE, which contains many phenolic acids and flavonoids, as an antiviral and anti-inflammatory agent.
